# Treatment of skeletal and non-skeletal alterations of Mucopolysaccharidosis type IVA by AAV-mediated gene therapy

**DOI:** 10.1038/s41467-021-25697-y

**Published:** 2021-09-09

**Authors:** Joan Bertolin, Víctor Sánchez, Albert Ribera, Maria Luisa Jaén, Miquel Garcia, Anna Pujol, Xavier Sánchez, Sergio Muñoz, Sara Marcó, Jennifer Pérez, Gemma Elias, Xavier León, Carles Roca, Veronica Jimenez, Pedro Otaegui, Francisca Mulero, Marc Navarro, Jesús Ruberte, Fatima Bosch

**Affiliations:** 1 Center of Animal Biotechnology and Gene Therapy, Bellaterra, Spain; 2 Department of Biochemistry and Molecular Biology, Universitat Autònoma de Barcelona, Bellaterra, Spain; 3 Centro de Investigación Biomédica en Red de Diabetes y Enfermedades Metabólicas Asociadas (CIBERDEM), Madrid, Spain; 4 Molecular Imaging Unit, Spanish National Cancer Research Center (CNIO), Madrid, Spain; 5 Department of Animal Health and Anatomy, School of Veterinary Medicine, Universitat Autònoma de Barcelona, Bellaterra, Spain

**Keywords:** Gene therapy, Metabolic disorders

## Abstract

Mucopolysaccharidosis type IVA (MPSIVA) or Morquio A disease, a lysosomal storage disorder, is caused by *N*-acetylgalactosamine-6-sulfate sulfatase (GALNS) deficiency, resulting in keratan sulfate (KS) and chondroitin-6-sulfate accumulation. Patients develop severe skeletal dysplasia, early cartilage deterioration and life-threatening heart and tracheal complications. There is no cure and enzyme replacement therapy cannot correct skeletal abnormalities. Here, using CRISPR/Cas9 technology, we generate the first MPSIVA rat model recapitulating all skeletal and non-skeletal alterations experienced by patients. Treatment of MPSIVA rats with adeno-associated viral vector serotype 9 encoding *Galns* (AAV9-*Galns*) results in widespread transduction of bones, cartilage and peripheral tissues. This led to long-term (1 year) increase of GALNS activity and whole-body correction of KS levels, thus preventing body size reduction and severe alterations of bones, teeth, joints, trachea and heart. This study demonstrates the potential of AAV9-*Galns* gene therapy to correct the disabling MPSIVA pathology, providing strong rationale for future clinical translation to MPSIVA patients.

## Abstract (web summary)

Mucopolysaccharidosis type IVA (MPSIVA) is a lysosomal storage disorder causing severe skeletal and non-skeletal alterations in patients. Here, the authors generate a MPSIVA rat model that mimics the disabling human pathology and develop an AAV9-Galns gene therapy to treat the disease.

## Introduction

Mucopolysaccharidosis type IVA (MPSIVA; Morquio A disease: OMIM #253000) is an autosomal recessive Lysosomal Storage Disorder (LSD) caused by the deficiency of N-acetylgalactosamine-6-sulfate sulfatase (GALNS, EC 3.1.6.4). The absence of this enzyme leads to the accumulation of the glycosaminoglycans (GAGs) Keratan Sulfate (KS) and Chondroitin-6-Sulfate (C6S)^1–3^. In MPSIVA patients, the pathological accumulation of undegraded KS and C6S, mainly in chondrocytes and extracellular matrix of cartilage, alters cartilage and bone development, leading to abnormal chondrogenesis and endochondral ossification^3,4^. Initial symptoms of the disease, including skeletal dysplasia, can be detected at young ages (mean age 2.1 years) in most patients^3,5^. Other non-skeletal manifestations are respiratory complications and valvular heart disease^2,6^.

Clinical pathology varies significantly from patient to patient depending on the mutation in the *GALNS* gene, ranging from a severe and rapidly progressive early-onset form to a slowly progressive later-onset form^5,7,8^. More than 30 genetic alterations are associated with attenuated phenotypes while over 100 mutations result in severe alterations in human patients^5,7,8^. Among them, patients bearing the most frequent and severe human missense mutation c.1156C>T, which leads to p.Arg386Cys in the GALNS protein^7,9^, show a clear correlation between genotype and phenotype^8^. In MPSIVA patients, death generally occurs among the second and third decade of life, although less severe forms of the disease present longer life expectancy^5,10^.

Treatment of MPSIVA disease is challenging due to the early onset of bone pathology and the difficulty of the treatment to reach isolated target tissues, such as avascular cartilage^11–13^. For most patients, control of the disease is merely symptomatic, aimed at improving their quality of life. The first approved treatment for Morquio A disease is an enzyme replacement therapy (ERT), a modified recombinant human GALNS (VIMIZIM^®^)^14,15^. Although treated patients show a reduction in urinary KS levels and improvement in the 6 min walking test, ERT is unable to correct skeletal and echocardiogram alterations, even when administered to young patients^14–18^. Since skeletal dysplasia is the main feature of Morquio A disease, new therapeutic approaches to treat bone and cartilage are needed. As other genetic disorders caused by deficiencies in secreted lysosomal enzymes, MPSIVA is also a candidate for gene therapy approaches. In vivo gene transfer with adeno-associated viral (AAV) vectors could overcome ERT-associated limitations, as transduced cells would allow constant production of active enzyme available to cross-correct cells. Moreover, AAV vectors have demonstrated high transduction efficiencies in vivo, long-term durability of therapeutic gene expression, and very good safety profiles in clinical studies^19–21^.

To develop gene therapies for MPSIVA disease, a limitation is that none of the existing MPSIVA mouse models develop the severe and disabling skeletal and articular pathology of Morquio A patients^22–24^. Treatment of MPSIVA mice with liver-directed AAV-based gene therapy resulted in increased secretion of GALNS and normalization of KS levels in plasma, liver, and lungs^25^. Nevertheless, because of the lack of skeletal dysplasia in MPSIVA mouse models, the therapeutic efficacy of this AAV gene therapy approach in the skeletal system could not be assessed^25^. The availability of animal models that reproduce human diseases is crucial for the development of efficacious therapies. For other diseases, including skeletal disorders, rat models show phenotypes closely resembling human pathological alterations^26–31^.

In the present study, to overcome the limitations of MPSIVA mouse models, we generated a rat model of MPSIVA using the CRISPR/Cas9 technology. This MPSIVA rat fully mimicked the severe skeletal and non-skeletal alterations found in human MPSIVA patients, thus representing the most relevant model to assess new therapeutic approaches for Morquio A disease. In this regard, in MPSIVA rats we have demonstrated that the use of AAV9 vectors in conjunction with the ubiquitous CAG promoter mediated widespread expression of *Galns* in bones, cartilage and ultimately in all key peripheral organs affected in Morquio A disease. We found that AAV9-*Galns* treatment corrected the severe whole-body alterations of MPSIVA rats, particularly skeletal dysplasia and cartilage deterioration. In this work, we demonstrate the effectiveness and long-term durability of the AAV9-*Galns* gene therapy to treat MPSIVA laying the foundation for future treatment of other diseases with skeletal involvement.

## Results

### Generation of a rat model of MPSIVA disease

A rat model of MPSIVA carrying the most frequent and severe human missense mutation c.1156C>T, that leads to p.Arg386Cys in the GALNS protein^7,9^, was generated using CRISPR/Cas9 technology (Supplementary Fig. 1a). Targeting of the *Galns* gene was confirmed by PCR and Sanger sequencing analyses (Supplementary Fig. 1b, c). Three selective backcrosses were performed between *Galns*^+/^^−^ and WT rats to eliminate CRISPR/Cas9-associated off-target events^32,33^. *Galns*^+/−^ rats, from two different lines (L1 and L2), had similar circulating GALNS activity (Supplementary Fig. 1d). *Galns*^−/−^ L1 and L2 rats showed indistinguishable phenotype (lacked GALNS activity, high hepatic KS content, reduced body weight (~50%), and naso-anal length (~20%)) (Supplementary Fig. 1e–h). L2 *Galns*^−/−^ rats were selected to further characterize the MPSIVA rat model. These rats showed undetectable levels of GALNS activity in all tissues analyzed and, as early as at 1 month of age, presented persistently high KS content in liver and serum, as well as a reduction of tibial length (Supplementary Fig. 1i–l), key features of Morquio A disease.

### AAV9-mediated widespread transduction of skeletal system

**Fig. 1:**
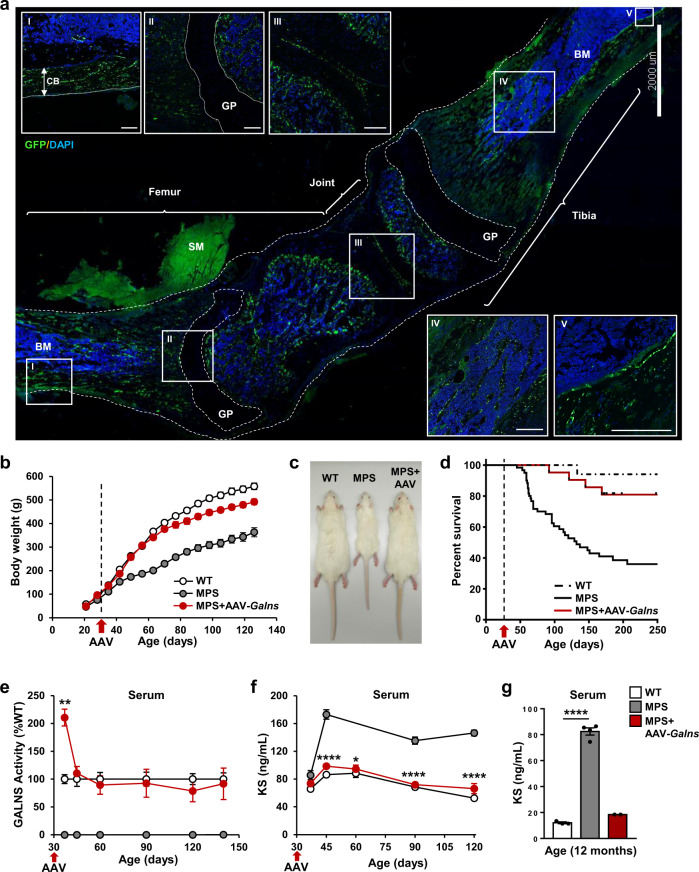
AAV9-*Galns* treatment corrects MPSIVA pathology. **a** Widespread transduction of femur and tibia after intravascular administration of 6.67 × 10^13^ vg/kg AAV9-*GFP* vectors to 4-weeks-old wild-type (WT) rats. Two weeks later, GFP signal was detected in compact bone (CB) (I), areas surrounding growth plate (GP) (II), joint (III), trabecular bone (IV), and endosteum (V). *n* = 3 animals/group. Scale bars: 2000 µm; insets, 300 µm. BM bone marrow, SM skeletal muscle. Afterward, 4-weeks-old MPSIVA male rats were treated with 6.67 × 10^13^ vg/kg AAV9-*Galns* vectors. **b** Body weight of WT (*n* = 21), MPSIVA untreated (*n* = 26), and AAV9-*Galns*-treated (*n* = 20) rats. **c** Representative image of 2-months-old WT, untreated and AAV9-*Galns*-treated MPSIVA rats. **d** Kaplan–Meier survival analysis, WT (*n* = 17), untreated (*n* = 76), and AAV9-*Galns*-treated MPSIVA (*n* = 32) rats. **e** Circulating GALNS activity of WT (*n* = 5), untreated (*n* = 5) and AAV9-*Galns*-treated MPSIVA (*n* = 18) rats. WT activity was set to 100%, which ranged from 94 to 133 nmol/17 h/mg. **f**, **g** Serum KS levels at different ages of WT (*n* = 4), untreated (*n* = 5) and AAV9-*Galns*-treated MPSIVA (*n* = 7) rats (**f**) and of 1-year-old WT (*n* = 3), untreated (*n* = 4) and AAV9-*Galns*-treated MPSIVA (*n* = 2) rats (**g**). All results are shown as mean ± SEM and *P*-values of one-way Anova test are indicated (95% confidence interval). *P < 0.05, **P < 0.01, and ****P < 0.0001 *vs*. untreated MPSIVA rats. Source data are provided as a Source Data file.

The major challenge facing a gene therapy for this disease is to achieve widespread transduction of the skeletal system to increase the local concentration of GALNS, since bone dysplasia is the most serious alteration affecting MPSIVA patients^3,34^. When AAV9 vectors encoding green fluorescent protein under the control of the CAG promoter (AAV9-*GFP)* were administered intravascularly to 4-weeks-old male rats, at a dose of 6.67 × 10^13^ vg/kg, high transduction of femur and tibia was observed (Fig. 1a). The GFP-positive signal was detected in osteoblast-rich areas surrounding femoral and tibial growth plate (GP) and endosteum, in the inner cells of the compact bone, and in articular cartilage (Fig. 1a). Moreover, high levels of GFP expression were also noted in all bones analyzed (femur, tibia, forelimb bones, sternum, ribs, and vertebrae) and key peripheral organs in Morquio A disease (Supplementary Fig. 2a, b).

Similarly, when MPSIVA rats were treated with AAV9 vectors encoding optimized rat *Galns* coding sequence under the control of the CAG promoter (AAV9-*Galns*) (6.67 × 10^13^ vg/kg) at 4 weeks of age, widespread vector biodistribution (vector genomes/diploid genome; vg/dg) throughout skeletal and non-skeletal tissues was observed (Supplementary Fig. 2c, d). All bones analyzed (long bones: femur, tibia, fibula, and humerus; flat bone: scapula; irregular bone: vertebrae; sesamoid bone: kneecap) as well as liver, heart, white adipose tissue (WAT), and lung were efficiently transduced (Supplementary Fig. 2c, d), indicating the potential of AAV9 vectors to treat MPSIVA pathology.

### Correction of MPSIVA pathology by AAV9-*Galns* treatment

**Fig. 2:**
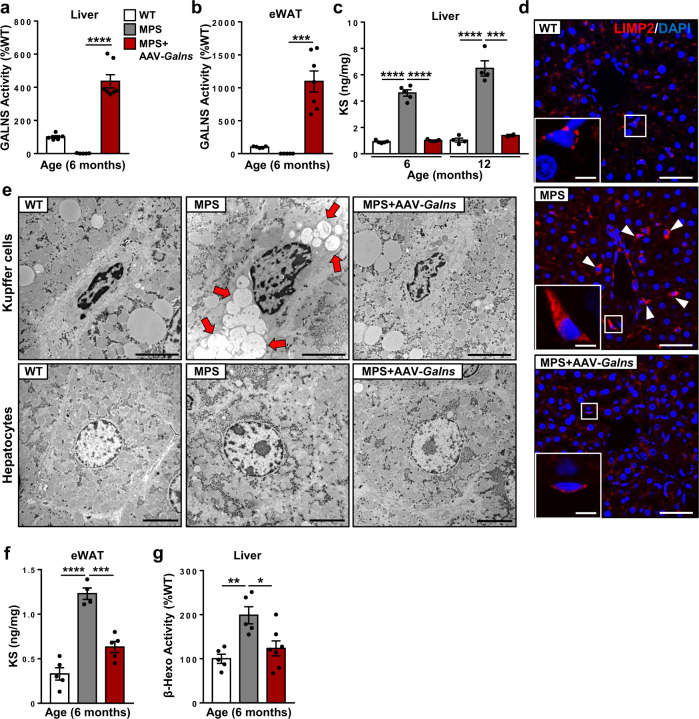
Treatment with AAV9-*Galns* vectors reverts liver and WAT alterations. MPSIVA rats were treated intravascularly with 6.67 × 10^13^ vg/kg AAV9-*Galns* vectors at 4 weeks of age. **a**, **b** GALNS activity in liver (**a**) and epididymal white adipose tissue (eWAT) (**b**) of 6-months-old WT (*n* = 5), untreated (*n* = 5) and AAV9-*Galns*-treated (*n* = 7) MPSIVA rats. WT activity was set to 100%, corresponding to 70.16 ± 5.98 (liver) and 62.77 ± 3.59 (eWAT) nmol/17 h/mg. **c** Hepatic KS content in 6- and 12-months-old WT (n = 4,5 respectively) and MPSIVA untreated (*n* = 4, 5, respectively) and AAV9-*Galns*-treated (*n* = 2, 5, respectively) rats. **d** LIMP2 immunohistochemistry of liver sections from the same cohort as in **a**. Untreated MPSIVA rats showed signs of lysosomal distension, which was absent in AAV9-*Galns*-treated MPSIVA rats. Scale bars: 50 µm; insets, 10 µm. **e** Ultrastructural analysis of Kupffer cells and hepatocytes revealed large electrolucent vacuoles (arrows) in Kupffer cells from untreated MPSIVA rats, that were absent in WT and in treated MPSIVA rats. No storage pathology was observed in hepatocytes. *n* = 2 animals/group. Scale bars, 5 µm. **f** KS content in eWAT from 6-months-old WT (*n* = 5), untreated (*n* = 4), and AAV9-*Galns*-treated (*n* = 5) MPSIVA rats. **g** β-Hexosaminidase activity in the same cohorts as in **a**. WT activity was set to 100%, corresponding to 931.97 ± 97.81 nmol/17 h/mg. All results are shown as mean ± SEM and *P*-values of one-way Anova test are indicated (95% confidence interval). *P < 0.05, **P < 0.01, ***P < 0.001, and ****P < 0.0001 *vs*. untreated MPSIVA rats. Source data are provided as a Source Data file.

Short stature is a hallmark of MPSIVA patients^5^. Although MPSIVA male rats showed similar body weight to WT rats during the first month of age, an important reduction in body weight and size was observed afterward (Fig. 1b, c and Supplementary Fig. 2e). WT rats showed a fast increase in body weight during the first 2 months of age. Reduction of MPSIVA rat body weight gain was parallel to decreased lifespan and ~30% of these rats died when 2-months-old (Fig. 1d). To prevent these alterations, MPSIVA rats should be treated early, by the first month of age. Treatment of 4-weeks-old MPSIVA rats with AAV9-*Galns* (6.67 × 10^13^ vg/kg) led to normalization of weight gain, naso-anal length, and survival rate compared to non-treated MPSIVA animals (Fig. 1b–d and Supplementary Fig. 2e). AAV9-*Galns* administration also triggered long-term normalization of circulating GALNS activity and serum KS levels (up to 12 months of age) (Fig. 1e–g). This probably resulted from increased *Galns* expression in liver and WAT (Supplementary Fig. 2f, g), leading to high GALNS activity in these organs (liver, >4-fold and WAT, >10-fold over WT levels) (Fig. 2a, b), both main tissues secreting proteins to circulation^35–37^, that persisted elevated long-term after treatment (Supplementary Fig. 2h, i). High GALNS activity led to long-term normalization of KS content in the liver, stored only in Kupffer cells, and WAT (Fig. 2c–f). Moreover, hepatic β-hexosaminidase activity, a lysosomal homeostasis biomarker^38–41^, was restored in AAV9-*Galns*-treated MPSIVA rats (Fig. 2g).

### AAV9-mediated *Galns* expression prevents skeletal alterations

**Fig. 3:**
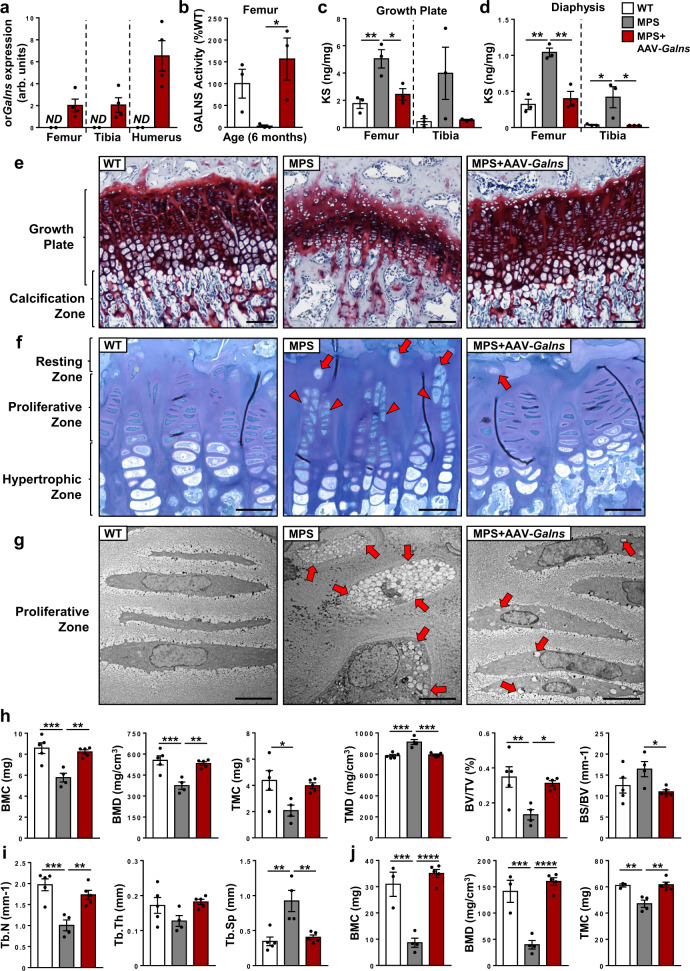
Prevention of bone and growth cartilage alterations after AAV9-*Galns* administration. Four-weeks-old MPSIVA rats were treated with 6.67 × 10^13^ vg/kg AAV9-*Galns* vectors. **a** Expression of optimized rat *Galns* (or*Galns)* in bones of 2-months-old WT (n = 2) and AAV9-*Galns*-treated MPSIVA (*n* = 4) rats. **b** Femoral GALNS activity in 6-months-old WT, untreated and AAV9-*Galns*-treated MPSIVA rats. WT activity was set to 100%, corresponding to 135.56 ± 44.81 nmol/17 h/mg (*n* = 3 animals/group). **c**, **d** KS content in the femoral and tibial growth plate (GP) and diaphysis in the same cohort as in **b**. **e** Histological analysis of humerus GP sections stained with Safranin O from 2-months-old WT (*n* = 5), untreated (*n* = 11), and AAV9-*Galns*-treated (*n* = 4) MPSIVA rats. Scale bars: 100 µm. **f** Toluidine-blue staining of semithin sections of tibial GP from 6-months-old WT, untreated and AAV9-*Galns*-treated MPSIVA rats. Treated rats showed a reduction of hypertrophic chondrocytes in resting (arrows) and proliferative (arrowheads) zones (*n* = 3 animals/group). Scale bars, 50 µm. **g** Ultrastructural analysis by TEM of tibial proliferative chondrocytes of the same cohort as in **f**. AAV9-*Galns* treatment cleared most intracellular storage vesicles (arrows). Scale bars: 10 µm. **h**–**j** Analysis by μCT of bone mineral content (BMC), bone mineral density (BMD), tissue mass content (TMC), tissue mass density (TMD), bone volume/tissue volume (BV/TV), bone surface/bone volume (BS/BV), trabecular number (Tb.N), trabecular thickness (Tb.Th) and trabecular spacing (Tb.Sp) of femoral trabecular (**h**, **i**) bone of 2-months-old WT (*n* = 5), untreated (*n* = 4) and AAV9-*Galns*-treated (*n* = 5) MPSIVA rats, and compact (**j**) bone of 2-months-old WT (*n* = 3), untreated (*n* = 4) and AAV9-*Galns*-treated (*n* = 5) MPSIVA rats. All results are shown as mean ± SEM and *P*-values of one-way Anova test are indicated (95% confidence interval). *P < 0.05, **P < 0.01, ***P < 0.001, and ****P < 0.0001 *vs*. untreated MPSIVA rats. ND non-detected. Source data are provided as a Source Data file.

AAV9-*Galns* administration to 4-weeks-old MPSIVA rats increased rat *Galns* expression in bones, such as femur, tibia, and humerus (Fig. 3a and Supplementary Fig. 3a), confirming the potential of AAV9 vectors to transduce the skeletal system. As a result of both the endogenous production and uptake from circulation, bones from AAV9-*Galns*-treated MPSIVA rats showed GALNS activity levels higher than WT rats, which reverted KS accumulation in both GP and diaphysis (Fig. 3b–d and Supplementary Fig. 3b). Skeletal dysplasia in MPSIVA patients is mainly caused by the alteration in endochondral ossification resulting from abnormal chondrogenesis^2,3^. Although no differences were observed at 1 month of age, GP cartilage sections showed a marked reduction of both GP area and calcification zone in 2-months-old MPSIVA rats, evidencing alterations in ossification as rats aged (Fig. 3e and Supplementary Fig. 3c,d). In contrast, AAV9-*Galns*-treated MPSIVA rats presented indistinguishable GP morphology to WT rats, with large GP area and normal trabecular formation (Fig. 3e and Supplementary Fig. 3d). Analysis of tibial GP of MPSIVA rats showed hypertrophic chondrocytes in resting and proliferative zones, that were markedly decreased in AAV9-*Galns*-treated MPSIVA rats (Fig. 3f). This was further confirmed by transmission electron microscopy (TEM), evidencing a large reduction of electrolucent storage vacuoles in the proliferative chondrocytes of treated animals (Fig. 3g). These results were consistent with body size normalization observed in AAV9-*Galns*-treated MPSIVA rats (Fig. 1b, c). Micro-computed tomography (μCT) analysis of tibia and femur of 2- and 6-months-old AAV9-*Galns*-treated MPSIVA rats revealed similar trabecular and compact bone composition and structure and tibial length to WT rats (Fig. 3h–j and Supplementary Fig. 3e–j). Treated MPSIVA rats showed normal bone mineral density and content, as well as trabecular thickness, number, and space. In contrast, non-treated MPSIVA rats showed alteration in all bone parameters (Fig. 3h–j and Supplementary Fig. 3e–i). Altered circulating levels of Alkaline Phosphatase, a biomarker for osteoblast activity^42^, were also normalized in AAV9-*Galns*-treated MPSIVA rats (Supplementary Fig. 3k). Altogether, these results provide strong evidence of the potential of this gene therapy to treat bone pathology in MPSIVA disease.

### Treatment with AAV9-*Galns* vectors prevents dental alterations

**Fig. 4:**
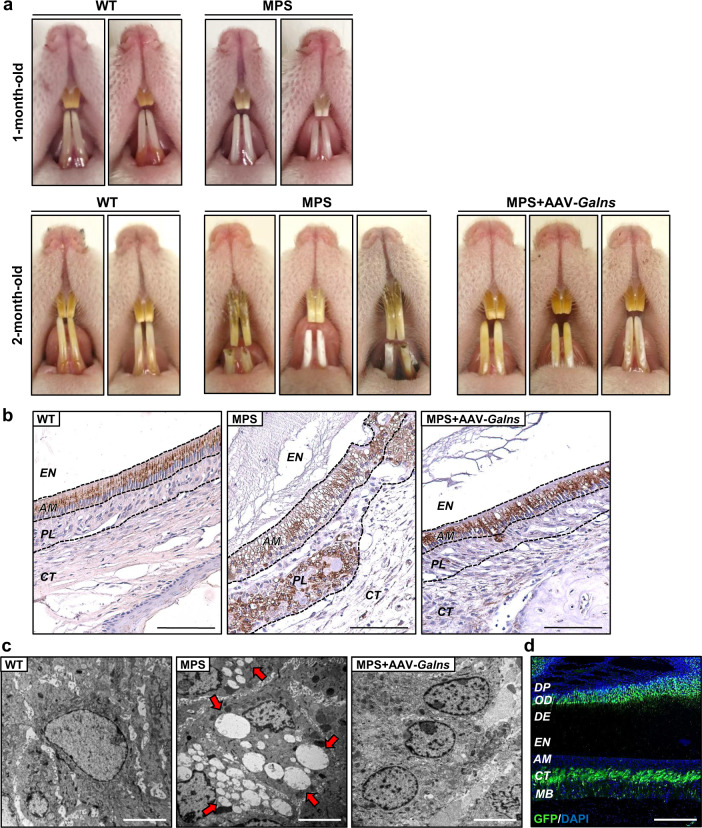
Treatment with *Galns*-encoding vectors counteracts dental pathology. MPSIVA rats were treated intravascularly with 6.67 × 10^13^ vg/kg AAV9-*Galns* vectors at 4 weeks of age. **a** Incisors from 1-month-old WT and MPSIVA rats presented similar morphology. At two months of age, incisors of MPSIVA rats presented malocclusion, dental fragility, and enamel hypoplasia, while incisors of MPSIVA treated rats were similar to those of WT rats. **b** LIMP2 immunohistochemistry of incisor in mandible sections from 2-months-old WT (*n* = 2), untreated (*n* = 3), and AAV9-*Galns-*treated (*n* = 2) MPSIVA rats. Untreated MPSIVA rats presented a marked lysosomal distension and vacuolization in ameloblast and papillary cell layers. Treatment of MPSIVA rats with AAV9-*Galns* prevented ameloblast lysosomal pathology. Scale bars: 100 µm. EN enamel, AM ameloblasts, PL papillary layer, CT connective tissue. **c** Ultrastructural analysis of incisors of 6-months-old rats. Ameloblast cells from incisors of untreated MPSIVA rats presented large electrolucent vacuoles (arrows), which were absent in AAV9-*Galns*-treated MPSIVA ameloblasts. *n* = 2 animals/group. Scale bars, 5 µm. **d** GFP immunostaining in incisors 2 weeks after administration of AAV9-*GFP* vectors (6.67 × 10^13^ vg/kg) to 4-weeks-old rats. *n* = 3 animals/group. DP dental pulp, OD odontoblasts, DE dentine, MB mandibular bone. Scale bars, 200 µm. Source data are provided as a Source Data file.

Dental alterations are common features of MPSIVA patients^2,43^. Although MPSIVA rats showed normal dental morphology at 1 month of age, dental malocclusion, fragility, and enamel hypoplasia were already evident in 2-month-old MPSIVA rats (Fig. 4a). In contrast, AAV9-*Galns*-treated MPSIVA rats presented intact incisors with normal occlusion and enamel (Fig. 4a). LIMP2 immunostaining of incisor in mandible sections from 2-months-old MPSIVA rats demonstrated marked lysosomal distension in enamel-producing ameloblast cells and papillary layer cells, resulting in enamel hypoplasia (Fig. 4a, b). AAV9-*Galns*-treatment corrected storage pathology in these cells (Fig. 4b). Ultrastructural analysis of MPSIVA rat incisors showed a high number of electrolucent storage vesicles in ameloblast cells. The absence of pathological lysosomal accumulation was noted after treatment with AAV9-*Galns* (Fig. 4c). Local GALNS production probably contributed to the correction of incisor alterations, since AAV9-*GFP* vectors efficiently transduced odontoblast and connective tissue layers near the ameloblast layer (Fig. 4d). Healthy enamel would protect patients from the high incidence of caries^2,43^.

### Treatment of articular cartilage pathology with AAV9-*Galns* gene therapy

**Fig. 5:**
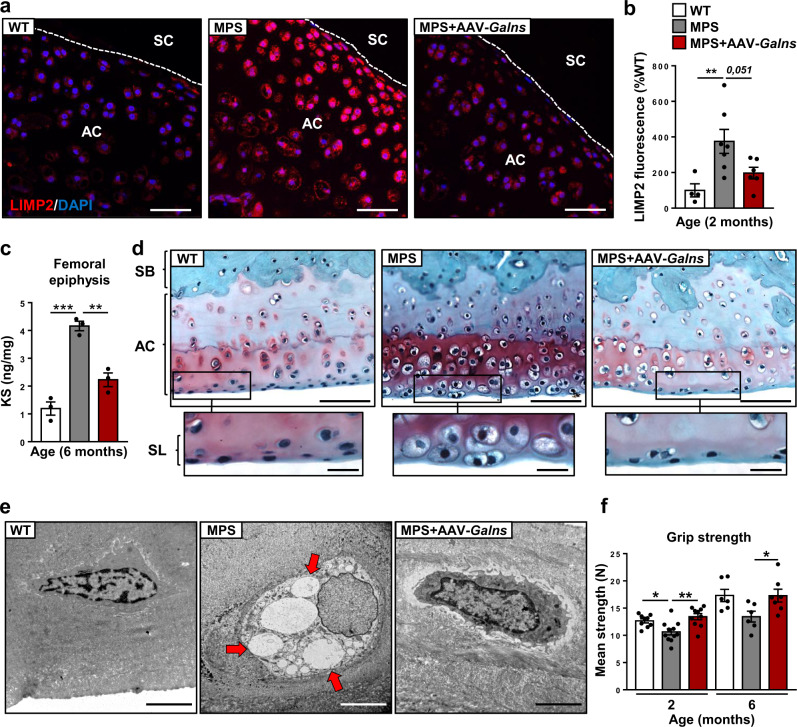
Treatment with *Galns*-encoding vectors of articular cartilage pathology. MPSIVA rats were treated intravascularly with 6.67 × 10^13^ vg/kg AAV9-*Galns* vectors at 4 weeks of age. **a** LIMP2 immunohistochemical analysis of humeral articular cartilage sections from 2-months-old WT, untreated and AAV9-*Galns*-treated MPSIVA rats. Articular chondrocytes from MPSIVA rats presented severe lysosomal distension that was markedly reduced after AAV9-*Galns* treatment. Scale bars: 50 µm. AC articular cartilage, SC synovial cavity. **b** Quantification of LIMP2 mean fluorescence in articular cartilage of 2-months-old WT (*n* = 4), untreated (*n* = 7) and AAV9-*Galns*-treated (*n* = 6) MPSIVA rats. **c** KS content in the distal femoral epiphysis of 6-months-old WT, untreated and AAV9-*Galns*-treated MPSIVA rats (*n* = 3 animals/group). **d** Safranin O staining of histological sections of humerus articular cartilage of 6-months-old WT, untreated and AAV9-*Galns*-treated MPSIVA rats (*n* = 3 animals/group). Scale bars, 50 µm; insets, 20 µm. SB subchondral bone, SL superficial layer. **e** Ultrastructural analysis of SL from tibial articular cartilage of 6-months-old WT, untreated and AAV9-*Galns*-treated MPSIVA rats. Large electrolucent vacuoles (arrows) were detected in chondrocytes of untreated MPSIVA rats, that were absent after AAV9-*Galns* treatment. *n* = 2 animals/group. Scale bars, 5 µm. **f** Grip strength analysis of 2- and 6-months-old WT (*n* = 9, 6, respectively), untreated (*n* = 11, 6, respectively) and AAV9-*Galns-*treated (*n* = 10, 7, respectively) MPSIVA rats. All results are shown as mean ± SEM and *P*-values of one-way Anova test are indicated (95% confidence interval). *P < 0.05, **P < 0.01, and ***P < 0.001 vs. untreated M*P*SIVA rats. Source data are provided as a Source Data file.

KS accumulation alters articular cartilage in MPSIVA patients leading to severe osteoarthritis that, in time, will require orthopedic surgery^3,44,45^. In MPSIVA rats at 4 weeks of age, analysis of tibial cartilage sections with both Safranin O staining and LIMP2 immunostaining already demonstrated increased chondrocyte hypertrophy (about 2-fold) compared with WT rats, indicating early storage pathology (Supplementary Fig. 4a–c). At 8 weeks of age, exacerbated LIMP2 signal was observed in articular cartilage sections from MPSIVA rats, consistent with progressive KS accumulation and lysosomal distension (Fig. 5a, b). AAV9-*Galns* treatment of MPSIVA rats resulted in marked correction of the storage pathology in articular cartilage (Fig. 5a, b). In agreement, analysis of distal femoral epiphysis in MPSIVA rats showed increased KS levels (~4-fold), that were significantly reduced after AAV9-*Galns* treatment (Fig. 5c). Histological analysis of humerus cartilage sections from 6-months-old MPSIVA rats, stained with Safranin O, evidenced an osteoarthritic phenotype with a high number of hypertrophic chondrocytes in the most superficial layer of the articular cartilage (Fig. 5d). AAV9-*Galns* treatment was able to reduce the number of hypertrophic chondrocytes in this layer (Fig. 5d). Ultrastructural analysis of chondrocytes from the superficial layer of tibial articular cartilage revealed a lack of electrolucent storage vesicles in AAV9-*Galns*-treated MPSIVA rats compared with non-treated animals (Fig. 5e). Similarly, AAV9-*Galns* treatment reduced chondrocyte hypertrophy and ultimately prevented articular cartilage loss in knee joints 11 months after vector administration to MPSIVA rats (Supplementary Fig. 4d, e). This agreed with the normalization of matrix metalloproteinase 13 expression in articular cartilage of AAV9-*Galns*-treated rats, contributing to osteoarthritis prevention (Supplementary Fig. 4f). Moreover, the lysosomal distension observed in the synovial membrane was also normalized by AAV9-*Galns* administration (Supplementary Fig. 4g). Consistent with joint alterations, MPSIVA rats already showed reduced grip strength at 2 months of age (Fig. 5f). AAV9-*Galns*-treated MPSIVA rats behaved like WT rats (Fig. 5f), suggesting that this gene therapy would ameliorate osteoarthritic alterations of MPSIVA disease.

### AAV9-*Galns* treatment of trachea and heart pathology

**Fig. 6:**
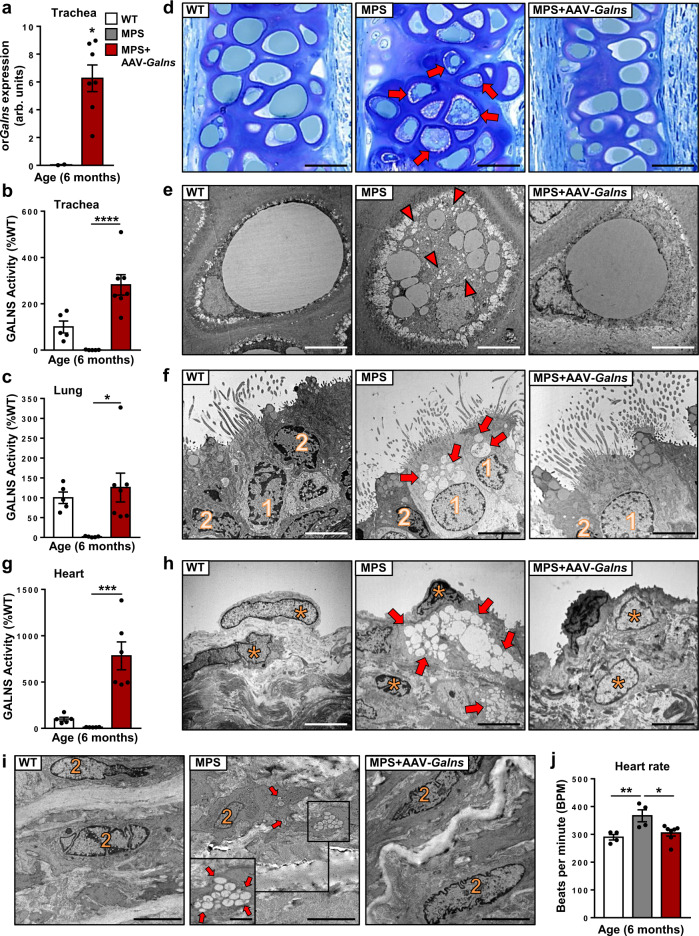
Correction of tracheal and cardiac pathology in AAV9-*Galns*-treated MPSIVA rats. Analysis of trachea and heart of 6-month-old WT and MPSIVA rats, untreated or treated with AAV9-*Galns* (6.67 × 10^13^ vg/kg) at 4 weeks of age. **a** Expression of or*Galns* in the trachea of WT (*n* = 2) and AAV9-*Galns*-treated MPSIVA (*n* = 7) rats. **b**, **c** GALNS activity in the trachea (**b**) and lung (**c**) of 6-months-old WT (*n* = 5), untreated (*n* = 5), or AAV9-*Galns-*treated (*n* = 7) MPSIVA rats. WT GALNS activity was set to 100%, corresponding to 173.26 ± 44.43 (trachea) and 137.63 ± 19.76 (lung) nmol/17 h/mg. **d** Toluidine-blue staining of semithin sections of trachea from untreated MPSIVA rats revealed a high number of small lipid droplets (arrows) in chondrocytes, that were not observed in trachea from AAV9-*Galns-*treated MPSIVA rats (*n* = 3 animals/group). Scale bars, 20 µm. **e** Ultrastructural analysis of tracheal chondrocytes from MPSIVA rats showed small electrolucent storage vesicles (arrowheads) and multiple lipid vesicles. AAV9-*Galns*-treated MPSIVA and WT chondrocytes presented a large lipid droplet. *n* = 2 animals/group. Scale bars, 5 µm. **f** Ultrastructural analysis of tracheal respiratory epithelium of untreated MPSIVA rats demonstrated abundant electrolucent vacuoles (arrows) in ciliated cells (1), that were absent after AAV9-*Galns* treatment. *n* = 2 animals/group. Scale bars, 5 µm. (2), goblet cells. **g** GALNS activity in heart of 6-month-old WT (*n* = 5), untreated (*n* = 5), or AAV9-*Galns-*treated (*n* = 6) MPSIVA rats. WT activity was set to 100%, corresponding to 42.24 ± 8.81 nmol/17 h/mg. **h**, **i** Ultrastructural analysis of untreated MPSIVA rats revealed abundant electrolucent vacuoles (arrows) in mitral valve cells (**h**) and smooth muscular fibers of aorta (**i**), that were not observed after AAV9-*Galns* treatment. *n* = 2 animals/group. Scale bars, 10 µm; inset, 1 µm. **j** Heart rate analysis of 6-month-old WT (*n* = 4), untreated (*n* = 4), or AAV9-*Galns-*treated (*n* = 7) MPSIVA rats. Results from **a** are shown as mean ± SEM and *P*-values of the unpaired two-tailed *t*-test are indicated. Results from **b**, **c**, **g**, **j** are shown as mean ± SEM, and *P*-values of the one-way Anova test are indicated (95% confidence interval). *P < 0.05, **P < 0.01, ***P < 0.001, and ****P < 0.0001 vs. untreated MPSIVA rats. Source data are provided as a Source Data file.

The most severe non-skeletal manifestations observed in MPSIVA patients are respiratory and cardiovascular complications, which are also the main cause of mortality^10,46^. Alterations in tracheal hyaline cartilage, respiratory epithelium, and lamina propria were observed in MPSIVA rats. AAV9-*Galns* treatment resulted in high levels of *Galns* expression and GALNS activity in the trachea and lung, leading to normalization of storage pathology in chondrocytes, ciliary cells, and fibroblasts (Fig. 6a–f and Supplementary Fig. 5a), reverting the respiratory tract alterations of MPSIVA animals.

Similarly, high levels of *Galns* expression and activity were observed in heart of MPSIVA rats after AAV9-*Galns* administration (Supplementary Fig. 5b and Fig. 6g). However, the main cardiac abnormalities of Morquio patients are valvular^2,47,48^. In agreement, compared to WT rats, MPSIVA rats presented no differences in heart weight, LIMP2 immunostaining of myocardial sections, and KS content in the myocardium, consistent with no alterations in myocardial fibers (Supplementary Fig. 5c–f). In contrast, and similarly to patients, TEM analysis demonstrated storage alterations in the mitral valve of MPSIVA rats (Fig. 6h). AAV9-*Galns* treatment resulted in the normalization of storage pathology of mitral valve cells (Fig. 6h). Moreover, lysosomal distension of smooth muscle fibers of aorta wall sections was also observed after LIMP2 immunostaining in MPSIVA rats, which was normalized in AAV9-*Galns*-treated MPSIVA rats (Supplementary Fig. 5g). Correction of storage pathology was further confirmed after ultrastructural analysis of aorta fibers in MPSIVA rats treated with AAV9-*Galns* that showed a lack of electrolucent vacuoles (Fig. 6i). The valvular dysfunction and aorta wall alterations in MPSIVA rats may have contributed to increasing heart rate (Fig. 6j), which is also altered in patients^48^. Treatment of MPSIVA rats with AAV9-*Galns* was also able to normalize heart rate (Fig. 6j).

## Discussion

Treatment of diseases with skeletal involvement is challenging because of the early onset of bone growth impairment in pediatric patients and the difficulty to treat isolated target tissues, as avascular cartilages^11–13^. MPSIVA is mainly characterized by progressive skeletal dysplasia, early cartilage deterioration, and cardiorespiratory alterations that lead to life-threatening complications^2,3^. The first key achievement of this study is the generation and characterization of a rat model recapitulating all these severe skeletal and non-skeletal alterations afflicting MPSIVA patients^2,3,43,46–49^. None of the existing MPSIVA mouse models reproduce the severity of the human disease, especially skeletal dysplasia and early cartilage deterioration^22–24^. Similar to MPSIVA rats, for many other diseases affecting bone, rat models can develop pathological alterations resembling those found in human patients. In this regard, an osteocalcin-null rat model demonstrated that the rat may be a more appropriate animal model than the mouse to investigate osteocalcin function in human skeletal diseases and to develop new therapeutic approaches for osteoporosis and osteoarthritis^28^. Similarly, osteosarcomas found in humans with *Tp53* mutations, but not found in mouse *Tp53* mutants, can also be detected in the rat *Tp53* models, providing a new animal model for the study of pediatric osteosarcomas^29^. Moreover, the rat provides a unique possibility to study arthritis induced by oil adjuvants, since adjuvants alone do not induce models of rheumatoid arthritis in other animal species, such as the mouse^30^. In addition, for MPSVI disease, the MPSVI rat model develops a more severe bone phenotype than the MPSVI mouse model^26,50–52^, in agreement with our MPSIVA rat model. All these studies highlight the translational impact of the rat models in skeletal-related diseases. Likewise, also for other rare genetic diseases, such as Duchene muscular dystrophy^27^, the rat model outperforms the mouse model of the disease.

The MPSIVA rat model was essential to assess the efficacy and long-term durability of the AAV9-*Galns* gene therapy to treat the whole plethora of pathological alterations of MPSIVA disease, including skeletal system and cartilage alterations. The therapeutic benefit resulted from AAV9-*Galns* vector-mediated widespread biodistribution after intravascular administration, together with the use of the ubiquitous CAG promoter that enabled the local increase of *Galns* expression in the main peripheral tissues affected in MPSIVA disease. Noticeably, AAV9 vectors were also able to transduce the rat skeletal system. The efficient bone and cartilage transduction mediated by AAV9 vectors were clearly demonstrated after administration of AAV9-*GFP* vector, which transduced osteoblasts-rich areas in compact and trabecular bone tissues. Osteoblasts produce mineral bone matrix and differentiate to osteocytes that remain surrounded by the matrix^53,54^. Osteocytes are long-lived cells that regulate bone metabolism and function^55^. The presence of GFP^+^ cells inside compact bone indicated that AAV9-*GFP* was able to efficiently transduce these cell types. AAV9-*Galns* administration also resulted in the expression of *Galns* in bones, which led to long-term normalization of KS storage in diaphysis and GP and to normal endochondral ossification of both compact and trabecular bone of tibia and femur. In addition, the administration of AAV9-*Galns* also corrected the severe progressive chondrocyte hypertrophy and cartilage loss. After AAV9-*Galns* transduction, the low chondrocyte turnover^56–58^ may have also contributed to long-term benefit, since the normalization of the severe joint alterations of MPSIVA rats were observed 1 year after treatment. None of the previously developed therapeutic approaches for MPSIVA was able to demonstrate correction of skeletal dysplasia using the existing mouse models^13,25,59^. The lack of efficacy of ERT to correct bone growth impairment in MPSIVA patients highlights the relevance of using animal models that closely reproduce the human disease to demonstrate therapeutic benefit before moving to the clinic^14,16^.

Moreover, uptake of GALNS protein from circulation could have contributed to correct bone and cartilage pathology in AAV9-*Galns*-treated MPSIVA rats. This treatment also resulted in extensive hepatic and WAT transduction and ultimately in high and long-term GALNS activity after gene transfer. Liver and WAT have been reported to efficiently secrete proteins of interest to the bloodstream upon AAV-mediated genetic engineering^35–37,60,61^. Thus, liver and WAT contributed to increase circulating GALNS activity in the treated rats resulting in long-term normalization of KS accumulation in these organs and serum. Transduction of these secretory organs also contributed to cross-correct cells from skeletal and non-skeletal tissues. Given that white adipocytes have a low turnover rate^62,63^, AAV-mediated transduction of WAT would overcome the loss of therapeutic efficacy imposed by hepatocyte replication in young animals^61,64–69^. Nevertheless, increased GALNS serum levels were not the only source of GALNS activity to treat bone, cartilage, trachea, and heart pathology since the *Galns* gene was also locally expressed in all these tissues, leading to long-term normalization of lysosomal GAG storage.

In contrast, specific genetic engineering of the liver to secrete the GALNS protein after treatment with AAV8-TBG-h*Galns* and AAV8-TBG-D8-h*Galns* vectors, solely ameliorated the mild phenotype of two MPSIVA mouse models^25^. Although these treatments corrected KS levels in serum, liver, and lung, no data about levels of KS storage throughout the skeletal system were shown in this study^25^. At 4 weeks of age, intravenous administration of AAV8-TBG-h*Galns* vectors at a dose similar to one of AAV9-CAG-*Galns* vectors used to treat MPSIVA rats, failed to provide complete restoration of chondrocyte size in the growth plate and articular cartilage of both MPSIVA mouse models during the 12-weeks monitoring period^25^. Similarly, a liver-mediated gene therapy to treat MPSVI, a different MPS disease that also affects the skeletal system, with a very similar gene transfer strategy (AAV8-TGB-*Arsb*) did not mediate complete correction of the bone pathology in the animal models evaluated^26,69^. Following AAV9-CAG-*Galns* treatment, MPSIVA rats showed full correction of skeletal dysplasia, indicating the superior advantage of widespread transduction of skeletal and non-skeletal tissues to correct MPSIVA disease.

In MPSIVA, initial symptoms of the disease can be detected in very young patients (between 1 and 3 years of age, mean age 2.1 years)^5^, suggesting that gene therapy treatment needs to be administered at a very early stage prior to the development of irreversible skeletal system alterations. Nevertheless, one of the main drawbacks of liver-directed AAV-mediated gene therapy approaches is the loss of therapeutic efficacy in pediatric patients (especially in very young individuals) due to increased hepatocyte cell division as patients grow older, and the episomal nature of the recombinant AAV genome^61,64–69^. This fact greatly narrows the population of patients than can obtain long-term benefit from liver-directed gene therapies. For MPSVI patients, only subjects of 4 years of age and older are eligible in the Phase I/II clinical trial using the liver-directed AAV8-TGB-h*ARSB* gene therapy (*ClinicalTrials.gov Identifier: NCT03173521*). Similarly, to prevent loss of transgene expression because of hepatocyte cell division, in all clinical trials performed up to date using liver-directed AAV-mediated gene therapy approaches to treat rare diseases, patients are older than 4 years^70^. In this regard, i.e., for Crigler Najjar, patients must be aged ≥10 years (ClinicalTrials.gov Identifier: NCT03466463), and for Hemophilia (ClinicalTrials.gov Identifier: NCT03001830) and Pompe disease (ClinicalTrials.gov Identifier: NCT04093349) the inclusion criteria establish that patients must be older than 18 years. Indeed, from a total of 30 ongoing clinical trials for liver-directed gene therapy to treat inborn errors of metabolism, 26 trials enroll patients older than 18 years^70^. For Morquio A patients, treatment at 4 years of age or older with a gene therapy approach may not be able to correct bone and cartilage alterations, especially for the severe and early-onset classical form of the disease. The results of our study point out the potential of the AAV9-CAG-*Galns* gene therapy to treat skeletal and cartilage pathology in MPSIVA disease at early stages, when the bone formation is very active.

In summary, this is the first study providing strong evidence of the long-term effects of AAV9-*Galns* treatment on the severe whole-body alterations, especially skeletal dysplasia and cartilage deterioration found in MPSIVA patients. These results support future clinical translation of this gene therapy to treat MPSIVA and create a therapeutic avenue for the treatment of bone diseases. Nevertheless, studies that investigate vector biodistribution and long-term safety of the approach in large animals (dogs and/or non-human primates), are required before moving the AAV9-CAG-*Galns*-mediated gene therapy to human patients.

## Methods

### Animals

A knock-in rat model of the MPSIVA disease was generated using CRISPR/Cas9 technology. To this end, the human and *Rattus norvegicus* GALNS protein sequences were aligned and the position of the human Arg386Cys mutation corresponded to position 388 in the rat protein sequence (Arg388Cys). Thus, the C>T single nucleotide change at the position 1156 of the human *Galns* coding sequence (CDS) is equivalent to the C at the position 1162 of the rat *Galns* CDS. Briefly, two specific RNA guides, gRNA1: 5′-CCC ATA TTT TAT TAC CGT GGC A-3′ and gRNA2: 5′-TAC CGT GGC AAC ACA CTG ATG G-3′, were designed to target exon 11 and drive the Cas9 double-strand break close to the 1162 C genomic position. A single strand donor DNA sequence was designed to introduce both the 1162C>T missense mutation and a new *Mbo*II restriction site, required for genotyping analysis of the offspring. Two homology arms were also included in the donor DNA to enable homologous recombination with the rat *Galns* genomic sequence. The gRNA, donor DNA, and Cas9 mRNA were microinjected into the pronucleus of one-cell Sprague–Dawley (SD) rat embryos. For genotyping of the knock-in rats, common forward and reverse *Galns*-Fw: 5′-TGT GGT GTG ACC ATT CAC CT-3′ and reverse *Galns*-Rv: 5′-TTT GTC AGC CCC ATT TCC TA-3′ primers were used (Supplementary Table 1). PCR reaction generated a 915 bp amplicon, a sequence encompassing the point mutation, that was further digested with *Mbo*II restriction enzyme generating: three fragments of 697, 142, and 76 bp in the WT allele; and four fragments of 381, 316, 142, and 76 bp in the *Galns* targeted allele. The resulting knock-in rats with the targeted allele were confirmed by Sanger sequencing of PCR amplicons using an ABI 3130xl genetic analyzer (ThermoFisher Scientific). Heterozygous knock-in male rats (*Galns*^*+/−*^) were backcrossed with WT rats for three generations (F1, F2, and F3) to segregate and eliminate possible CRISPR/Cas9-associated off-target events. Homozygous mutant rats (*Galns*^*−/*−^ or MPSIVA rats) were obtained by mating heterozygous littermates of the F3 generation. Male rats were fed *ad libitum* with a standard diet (Teklad, Envigo) and maintained under a light-dark cycle of 12 h (lights on at 8:00 A.M.) and stable temperature (22 °C ± 2). All experimental procedures were performed following the Federation of Laboratory Animal Science Associations (FELASA) guidelines and the ethical and legal requirements concerning the use of animals in research from Generalitat de Catalunya, Spain, and the EU directives, and approved by the Ethics Committee for Animal and Human Experimentation of the Universitat Autònoma Barcelona (UAB).

### AAV vector production and administration

AAV expression cassettes were generated by cloning an optimized rat *N*-acetylgalactosamine-6-sulfate sulfatase (*Galns*) coding sequence (GeneArt; ThermoFisher Scientific) or the green fluorescent protein (GFP) cDNA under the control of the ubiquitous CAG promoter (hybrid of chicken β-actin promoter and CMV enhancer) into AAV backbone plasmids. The GFP construct also contained the WPRE element to enhance protein production. AAV vectors of serotype 9 were produced by triple transfection of HEK293 cells followed by a second-generation cesium chloride gradient-based purification method that renders vectors preps devoid of empty capsids^71^. Vectors were titered by real-time PCR and titers were expressed as vg/mL. For systemic administration, AAV vectors were diluted in PBS supplemented with 0.001% F68 Pluronic^®^ (ThermoFisher Scientific) and injected via tail vein. Male rats were treated with 6.67 × 10^13^ vg/kg of either AAV9-*Galns* or AAV9-*GFP* vectors.

### Sample collection

Rats were anesthetized by intraperitoneal injection of 80 mg/kg ketamine and 10 mg/kg xylazine. Blood was obtained by cardiac puncture. Afterward, animals were transcardially perfused with 100 mL of PBS to clear blood from tissues thus removing all traces of circulating GALNS. Tissues were dissected and either snap-frozen and stored at −80 °C or immersed in formalin for subsequent histological analysis. Bones were fixed at 4 °C in 10% buffered paraformaldehyde (Sigma-Aldrich).

### GFP fluorescence assay

For GFP fluorescence quantification, total protein extracts were obtained by mechanical disruption (T10 Ultra-Turrax^®^, IKA^®^-Werke GmbH & Co.) of several tissues in lysis buffer (50 mM Tris, 1% Nonident p40, 0,25% sodium deoxycholate, 150 mM NaCl, 1 mM EDTA in PBS, pH 7,4). After centrifugation, GFP released fluorescence was measured directly from the supernatants with a Synergy HTX fluorimeter (BioTek Instruments) at excitation *λ* = 488 nm and emission *λ* = 512 nm. RFU values were normalized by total protein content, quantified by BCA assay (ThermoFisher Scientific).

### Gene expression analysis

To determine *Galns* expression, total RNA was obtained from several tissues and bones with TriPure isolation reagent (Roche) using an RNeasy Mini Kit (Qiagen). Total RNA was reverse transcribed using the Transcriptor First Strand cDNA Synthesis kit (Roche). Optimized rat *Galns* (or*Galns*) expression was assessed through real-time quantitative PCR (qRT-PCR) in a LightCycler® 480 (Roche) using the TaqMan LightCycler® 480 Probe Master and or*Galns* specific primers, which do not recognize endogenous *Galns*. Forward primer, 5′-CGG AAG GTT CTA CGA AGA GTT C-3′; reverse primer, 5′-GTG CTG GGT CCT GAT GAA G-3′; probe: 56-FAM/AAC CTG ACC/ZEN/CAG CTG TAC CTG C/3IABkFQ (Supplementary Table 1). For *Mmp13* expression, total RNA was obtained from tibial articular cartilage using an RNeasy Micro Kit (Qiagen) and reverse transcribed. The expression was assessed by qRT-PCR using SYBR® Premix Ex Taq™ (Takara). Forward primer, 5′-TGG CGA CAA AGT AGA TGC TG-3′; reverse primer, 5′-TGG CAT GAC TCT CAC AAT GC-3′ (Supplementary Table 1). Values were normalized to the expression of the rat *Rplp0* gene. Forward primer, 5′-AAG CCA CAC TGC TGA ACA TG-3′; reverse primer, 5′-TGC TGC CAT TGT CAA ACA CC-3′ (Supplementary Table 1).

### Vector genome copy number

Tissue samples were digested overnight (ON) at 56 °C in 300 μL of Tissue Lysis Solution supplemented with Proteinase K (0.2 mg/mL). Bones were mechanically disrupted in water and afterward digested with proteinase K at 56 °C ON. Total DNA was isolated from supernatants with the MasterPureDNA Purification Kit (Lucigen). DNA was resuspended in distilled water and quantified using a NanoDrop ND‐1000 spectrophotometer (NanoDrop). Vector genome copy number in 20 ng of total DNA was determined by quantitative PCR using Light Cycler 480 Probes Master (Roche). Primers and probes were designed for a specific sequence within the optimized rat *Galns* cDNA. Forward primer, 5′-CGG AAG GTT CTA CGA AGA GTT C-3′; reverse primer, 5′-GTG CTG GGT CCT GAT GAA G-3′; probe: 56-FAM/AAC CTG ACC/ZEN/CAG CTG TAC CTG C/3IABkFQ (Supplementary Table 1). A reference standard curve was built from serial dilutions of a linearized plasmid bearing the CAG promoter and the optimized *Galns* cDNA spiked into 20 ng/µL of non-transduced rat genomic DNA.

### Enzyme activities

Soft tissues were sonicated in 200–500 µL of water and bones were mechanically disrupted in 500–1000 µL of homogenization buffer (25 mM Tris-HCl, pH 7.2 and 1 mM phenylmethylsulfonyl fluoride). Serum was analyzed unprocessed. GALNS and β-hexosaminidase activities were determined in clarified supernatants using the 4-methylumbelliferone-derived fluorogenic substrates^38,39^. Briefly, GALNS activity was assayed in 2 µg of protein extract or in 5 µl of serum, and incubated with 10 mM 4-Methylumbelliferyl β-d-Galactopyranoside-6-sulfate Sodium Salt (Toronto Research Chemicals) for 17 h at 37 °C, followed by a second incubation with β-galactosidase (Sigma-Aldrich) for 2 h at 37 °C. The activity of β-hexosaminidase was measured in 0.4 µg of total protein with 4-methylumbelliferyl *N*-acetyl-β-d-glucosaminide (Sigma-Aldrich) for 1 h at 37 °C. After stopping the reaction by increasing the pH, released fluorescence was measured with Synergy HTX fluorimeter (Biotek Instruments) at excitation *λ* = 360 nm and emission *λ* = 460 nm. Enzyme activities were normalized against the volume of serum or total protein content quantified by Bradford assay (Bio-Rad). Alkaline Phosphatase (ALP) was measured in 100 µL of serum samples by an enzymatic-colorimetric method (Beckman Coulter) using an AU 480 analyzer (Beckman Coulter).

### Keratan sulfate quantification

Soft tissues were weighed and digested at 56 °C overnight in water with proteinase K. Bones were weighed and mechanically disrupted in water and digested with proteinase K at 56 °C overnight. Extracts were clarified by centrifugation and filtration with 0.22 μm microporous membrane-containing filters (Ultrafree MC, Millipore). Clarified extracts and serum were digested with Keratanase II to obtain the keratan sulfate (KS)-derived disaccharides *Galβ1-4GlcNAc(6S)* and *Gal(6S)β1-4GlcNAc(6S)*, that were quantified by LC–MS/MS at the Division of Inborn Errors of Metabolism, Biochemical Diagnostic Center of the Hospital Clínic of Barcelona. KS content was normalized to wet tissue weight or serum volume.

### Histology and electron microscopy

Soft tissues were fixed for 12–24 h in 10% formalin, embedded in paraffin, and sectioned. Bones were fixed at 4 °C for 48 h in 10% buffered paraformaldehyde (Sigma-Aldrich) and progressively decalcified for 4 weeks (2-months-old rats) or 8 weeks (6- and 12-months-old rats) with ethylenediaminetetraacetic acid (EDTA). Once decalcified, bones were paraffin-embedded and sectioned for further histological studies. For GFP and LIMP2 immunohistochemistry, histological sections were incubated overnight at 4 °C with goat anti-GFP (Abcam, dil 1:300) or rabbit anti-LIMP2 (Novus Biologicals, dil 1:100) primary antibodies. Biotinylated Alexa Fluor^®^ 488 anti-goat IgG (Life technologies, dil 1:100) or Alexa Fluor^®^ 568 anti-rabbit IgG (Invitrogen, dil 1:100) were used as secondary antibodies. Hoechst (Sigma‐Aldrich) was used for nuclear counterstaining of fluorescent specimens. Images were obtained with a confocal Olympus Fluoview 1000 (Olympus Corp) or Leica SP5 (Leica Biosystems). Bone sections (3–4 µm) were treated with 1% Safranin O dye to stain cartilage (red) and bone tissue (blue). Growth plate (GP) Safranin O and heart LIMP2 areas were quantified using NIS Elements Advanced Research 2.20 software (Nikon Instruments) in 4–6 images (original magnification, ×10) per animal, obtained using an Eclipse 90i microscope (Nikon). LIMP2 mean fluorescence in articular cartilage was quantified as Mean Gray Value using Fiji/ImageJ 1.53c software in 4 images (original magnification, ×63) per animal, obtained using a Leica SP5 confocal microscope (Leica Biosystems).

For transmission electron microscopy (TEM) analysis, rats were euthanized by intraperitoneal ketamine/xylazine overdose. A small portion (~1 mm^3^) of each tissue was dissected and incubated at 4 °C for 2 h in 1 mL of 2.5% glutaraldehyde and 2% PFA, as a fixative. Specimens were postfixed in 1% osmium tetroxide, stained in aqueous uranyl acetate, and then dehydrated and embedded in epoxy resin. For semithin sections (800 nm), bone and tracheal samples were stained with toluidine-blue dye, and images were obtained with Nikon Eclipse 50i and 90i microscopes (Nikon). For TEM, ultrathin sections (600–800 Å) were stained using lead citrate and examined with a Hitachi H-7000 transmission electron microscope (Hitachi).

### Bone structural analysis

Bone composition and architecture were evaluated by micro-computed tomography (μCT) analysis. Rat tibiae and femur were fixed in neutral‐buffered formalin (10%) and scanned using the eXplore Locus CT scanner (General Electric) at 27 µm resolution. Compact bone was analyzed in 6 cm^3^ of the cortical femoral and tibial diaphysis and trabecular bone in 2.5 cm^3^ of proximal femoral and tibial epiphysis. Bone parameters were calculated with the MicroView 3D Image Viewer & Analysis Tool 2.2. The length of the tibia was measured from the medial condyle to the medial malleolus.

### Naso-anal length measurement

Rats were positioned supine and the distance between the most rostral part of the head and the most caudal part of the abdomen (base of the tail) was measured.

### Grip strength

The grip strength was measured as maximum tensile force using a grid and recorded with a rat Grip Strength Meter (Panlab) equipped with a sensor capacity of 25 Newtons and an accuracy of 0.1%. Three pulls of the Grip Strength Meter were averaged per rat.

### Heart rate analysis

Rats were anesthetized (2–4% isoflurane and O_2_ at 1 L/min). Afterward, electrodes were placed in the pectoral area to record beats per minute (bpm) for 2 min using an electrocardiograph.

### Statistical analysis and data processing

Results are expressed as mean ± SEM. Statistical comparisons were made using either unpaired two-tailed *t*-test or one-way analysis of variance (ANOVA). Multiple comparisons between control and treatment groups were made using the Dunnett post-test. Statistical significance was considered if *P* < 0.05. Kaplan–Meier curves were used to estimate survival and the log-rank test was used for comparisons. GraphPad Prism 7 software and Microsoft Excel 2013 were used to analyze data.

### Reporting summary

Further information on research design is available in the Nature Research Reporting Summary linked to this article.

## Supplementary information

Supplementary information

Reporting Summary

## Data Availability

The authors declare that all data supporting the findings of this study are available within the paper. Source data are provided with this paper.

## Source data

Source Data
