# Supplementary material for: Treatment of skeletal and non-skeletal alterations of Mucopolysaccharidosis type IVA by AAV-mediated gene therapy
Source: Nat Commun. 2021 Sep 9;12:5343. doi: 10.1038/s41467-021-25697-y (PMC8429698; doi:10.1038/s41467-021-25697-y)
Supplement: Supplementary file 1 — Supplementary information [file 41467_2021_25697_MOESM1_ESM.pdf]

Supplementary information

Treatment of skeletal and non-skeletal alterations of Mucopolysaccharidosis type IVA by AAV-mediated gene therapy

Joan Bertolin[1,2,6], Víctor Sánchez[1,2,6], Albert Ribera[1,2,3], Maria Luisa Jaén[1,2], Miquel Garcia[1,2,3], Anna Pujol[1,2], Xavier Sánchez[1,2], Sergio Muñoz[1,2,3], Sara Marcó[1,2], Jennifer Pérez[1,2], Gemma Elias[1,2], Xavier León[1,2,3], Carles Roca[1,2,3], Veronica Jimenez[1,2,3], Pedro Otaegui[1], Francisca Mulero[4], Marc Navarro[1,5], Jesús Ruberte[1,5] and Fatima Bosch[1,2,3*]

[1]Center of Animal Biotechnology and Gene Therapy, [2]Department of Biochemistry and Molecular Biology, Universitat Autònoma de Barcelona, Bellaterra, 08193. [3]Centro de Investigación Biomédica en Red de Diabetes y Enfermedades Metabólicas Asociadas (CIBERDEM), Madrid, Spain. [4]Molecular Imaging Unit, Spanish National Cancer Research Center (CNIO), Madrid, Spain. [5]Department of Animal Health and Anatomy, School of Veterinary Medicine, Universitat Autònoma de Barcelona, Bellaterra, 08193.
[6]These authors contributed equally: Joan Bertolin, Víctor Sánchez.
*Correspondence and requests for materials should be addressed to F.B. (e-mail: fatima.bosch@uab.es)

Supplementary Fig. 1

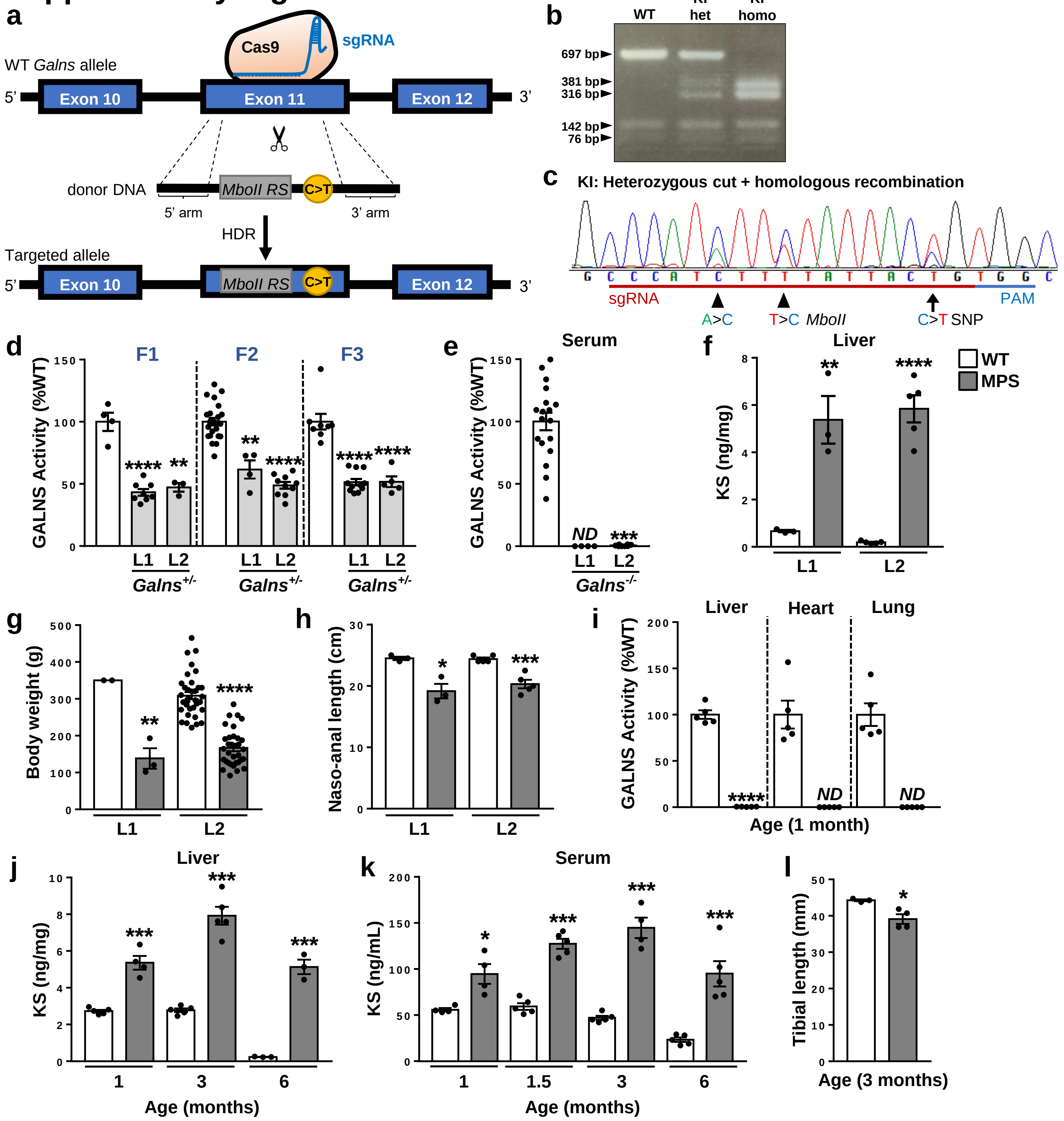

Supplementary Fig. 1. Generation of a MPSIVA rat model. a CRISPR/Cas9 strategy to generate *Galns* knock-in (KI) rats. Single guided RNAs (sgRNA) were designed to target exon 11 of rat *Galns* gene to introduce the C>T mutation and *Mbo*II restriction site by homology directed repair (HDR). **b** Rat genotyping by PCR analysis. (KI, knock-in). **c** Sanger sequencing confirmed C>T mutation (arrow) and *Mbo*II restriction site (arrowheads) in KI alleles. **d** Circulating GALNS activity of F1, F2 and F3 rat generations from two heterozygous KI (*Galns*$^{+/-}$) lines (L1 and L2). WT activity was set to 100%, corresponding to 56.09±4.09 (F1), 80.95±4.40 (F2) and 96.90±7.09 (F3) nmol/17h/mg. (n=3-24 animals/group). **e** Serum GALNS activity in 1-month-old WT and *Galns*$^{-/-}$ (MPSIVA) rats. WT activity was set to 100%, corresponding to 112.56±9.75 nmol/17h/mg. (WT, n=15; MPSIVA (L1), n=4; MPSIVA (L2), n=8). **f** Hepatic KS content in 3-month-old rats (n=3-5 animals/group). **g** Body weight of 2-month-old rats (WT (L1), n=2; MPSIVA (L1), n=3; WT (L2), n=35; MPSIVA (L2), n=33). **h** Naso-anal length of 3-month-old rats (n=3-5 animals/group). **i** GALNS activity in liver, heart and lung of 1-month-old rats. WT activity was set to 100%, corresponding to 344.10±42.63 (liver), 59.86±9.06 (heart) and 247.42±30.29 (lung) nmol/17h/mg. **j**,**k** KS content in liver (j) and serum (k) of WT and MPSIVA rats (n=4-5 animals/group). **l** μCT analysis of tibial length in 3-month-old WT and MPSIVA rats (n=3-4 animals/group). Results are shown as mean±SEM; *P<0.05, **P<0.01, ***P<0.001 and ****P<0.0001 *vs.* WT rats. *ND*, non-detected.

Supplementary Fig. 2

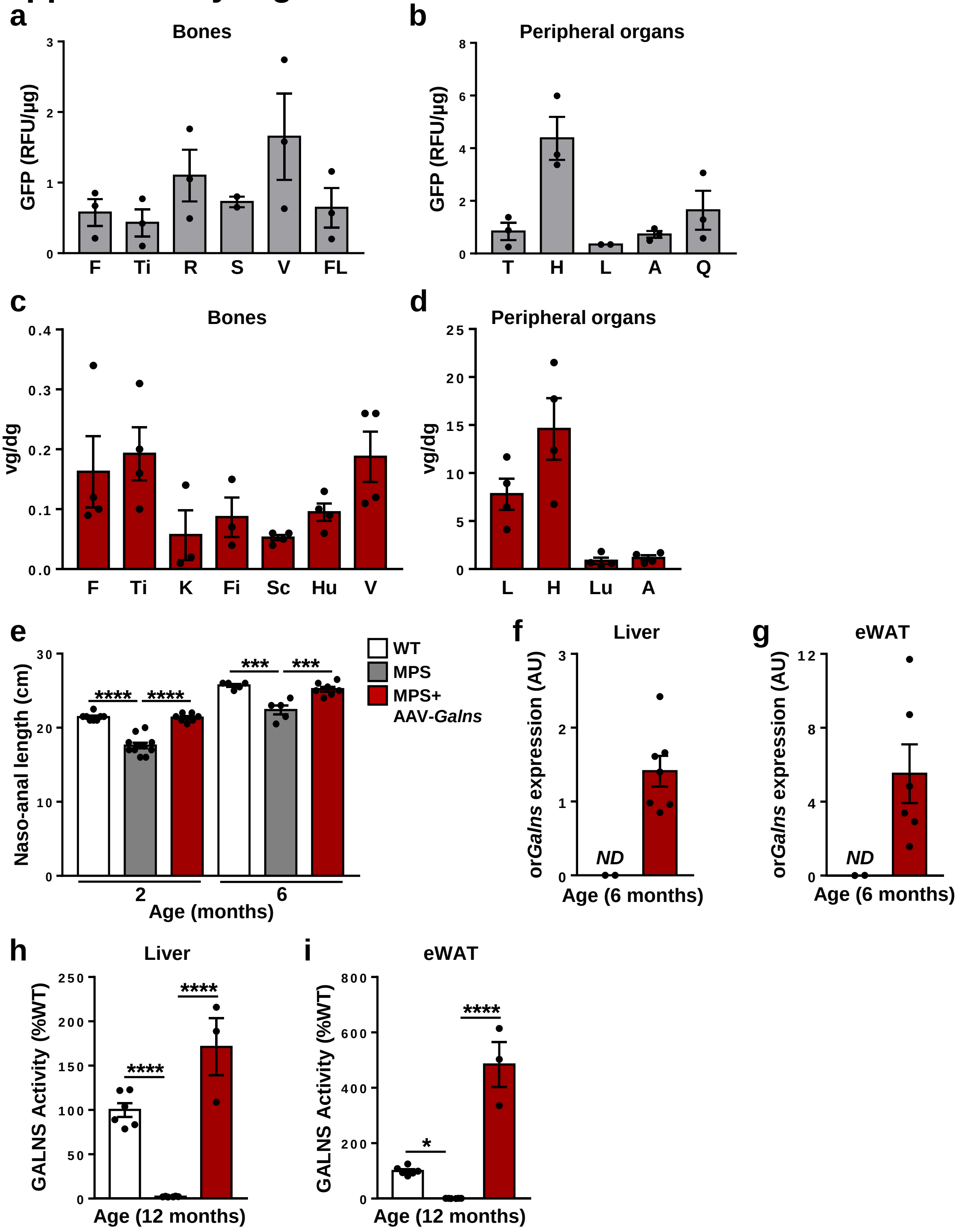

Supplementary Fig. 2. AAV9 vectors mediate widespread transduction of skeletal and non-skeletal tissues. a,**b** Intravascular administration of 6.67x10^{13} vg/kg AAV9-*GFP* vectors to 4-week-old rats resulted in high levels of GFP fluorescence in all bones (a) and peripheral organs (b) analyzed at 6 weeks of age (n=3 animals/group). **c-i** AAV9-*Galns* treatment (6.67x10^{13} vg/kg) of MPSIVA rats at 4 weeks of age counteracts naso-anal length, liver and epididymal white adipose tissue (eWAT) alterations. Vector genome copy number analysis showed efficient transduction of bones (c) and peripheral organs (d) in 2-month-old AAV9-*Galns*-treated MPSIVA rats. Naso-anal length (e) of 2- and 6-month-old WT, untreated and AAV9-*Galns*-treated MPSIVA rats (n=5-7 animals/group). Expression of optimized or*Galns* in liver (f) and eWAT (g) of WT (n=2) and AAV9-*Galns*-treated MPSIVA (n=6-7) rats. GALNS activity in liver (h) and eWAT (i) of 12-month-old WT, untreated and AAV9-*Galns*-treated MPSIVA rats. WT activity was set to 100%, corresponding to 82.90±4.48 (liver) and 102.28±6.29 (eWAT) nmol/17h/mg (n=3-6 animals/group). F, Femur; Ti, Tibia; R, Ribs; S, Sternum; V, Vertebrae; FL, Forelimb bones; K, Kneecap; Fi, Fibula; Sc, Scapula; Hu, Humerus; T, Trachea; H, Heart; L, Liver; A, eWAT; Lu, Lung; Q, Quadriceps. Results are shown as mean±SEM. *P<0.05, ***P<0.001 and ****P<0.0001 *vs.* untreated MPSIVA rats. *ND*, non-detected.

Supplementary Fig. 3

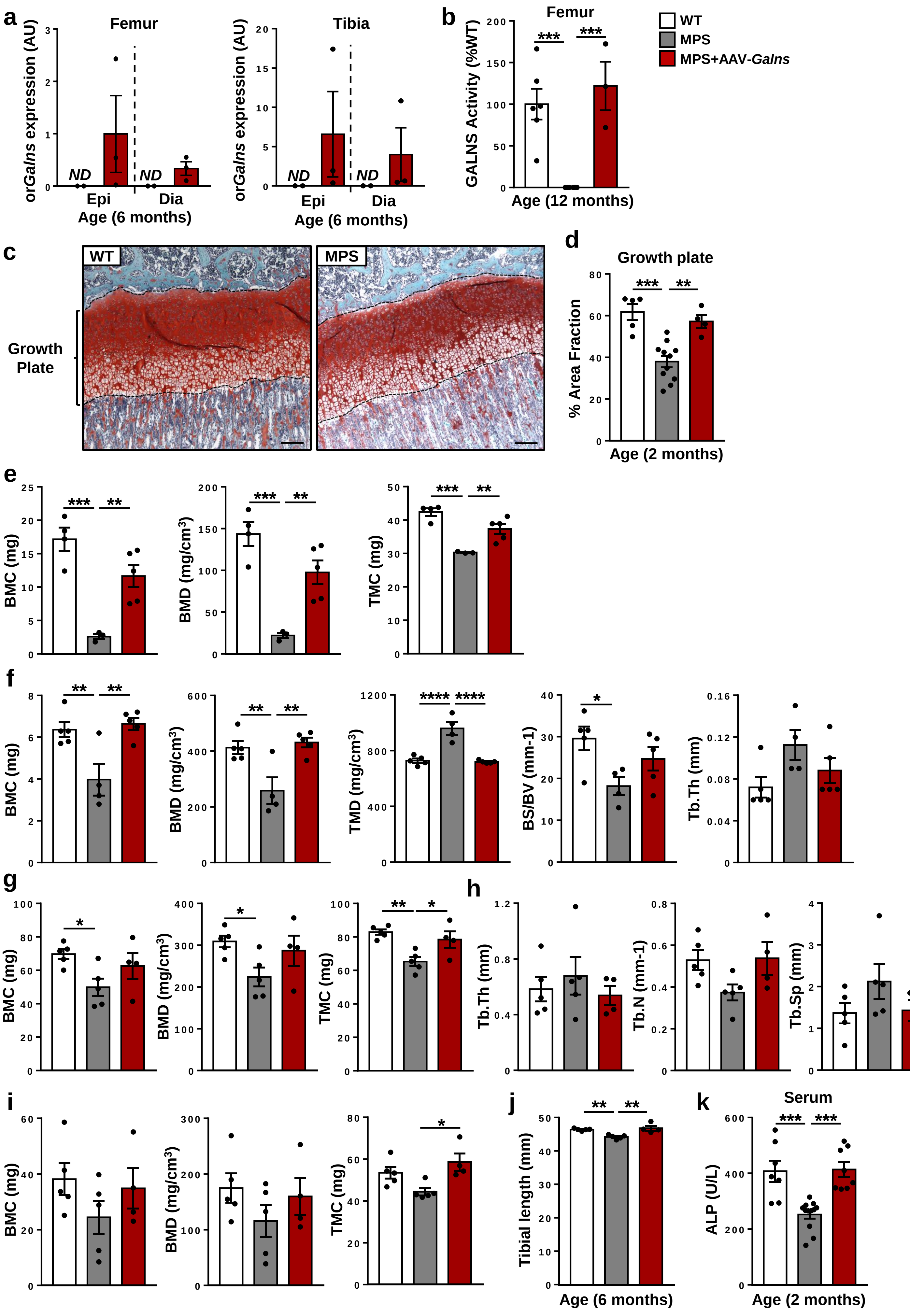

Supplementary Fig. 3. Correction of bone pathology in MPSIVA rats treated with AAV9-*Galns*. Four-week-old MPSIVA rats were treated with 6.67x10^{13} vg/kg AAV9-*Galns* vectors. **a** Expression of optimized rat *Galns* (or*Galns)* in femoral and tibial epiphysis and diaphysis of 6-month-old WT (n=2) and AAV9-*Galns*-treated MPSIVA (n=3) rats. **b** Femoral GALNS activity in 12-month-old WT, untreated and AAV9-*Galns*-treated MPSIVA rats. WT activity was set to 100%, corresponding to 1086.13±200.12 nmol/17h/mg. **c**, Histological analysis of tibial growth plate (GP) sections stained with Safranin O from 1-month-old WT and untreated MPSIVA rats (n=5 rats/group). Scale bars: 50µm. **d** Analysis of humerus GP area from 2-month-old WT (n=5), untreated (n=11) and AAV9-*Galns*-treated (n=4) MPSIVA rats (original magnification, x10). Histograms represent the quantification of the percentage of GP area per total bone area. **e**,**f** Analysis by μCT of bone mineral content (BMC), bone mineral density (BMD), tissue mass content (TMC), tissue mass density (TMD), bone surface/bone volume (BS/BV) and trabecular thickness (Tb.Th) of tibial compact (e) and trabecular (f) bone of 2-month-old WT, untreated and AAV9-*Galns*-treated MPSIVA rats (n=3-5 animals/group). **g-i** Analysis by μCT of BMC, BMD, TMC, Tb.Th, trabecular number (Tb.N) and trabecular space (Tb.Sp) of femoral compact (g) and trabecular (h) bone and tibial compact bone (i) of 6-month-old WT, untreated and AAV9-*Galns*-treated MPSIVA rats (n=4-5 animals/group). **j**, μCT analysis of tibial length in the same cohort as in (i). **k**, Serum Alkaline Phosphatase (ALP) of 2-month-old WT (n=7), untreated (n=12) and AAV9-*Galns*-treated (n=8) MPSIVA rats. Results are shown as mean±SEM. **P*<0.05, ***P*<0.01, ****P*<0.001 and *****P*<0.0001 *vs.* untreated MPSIVA rats. *ND*, non-detected.

Supplementary Fig. 4

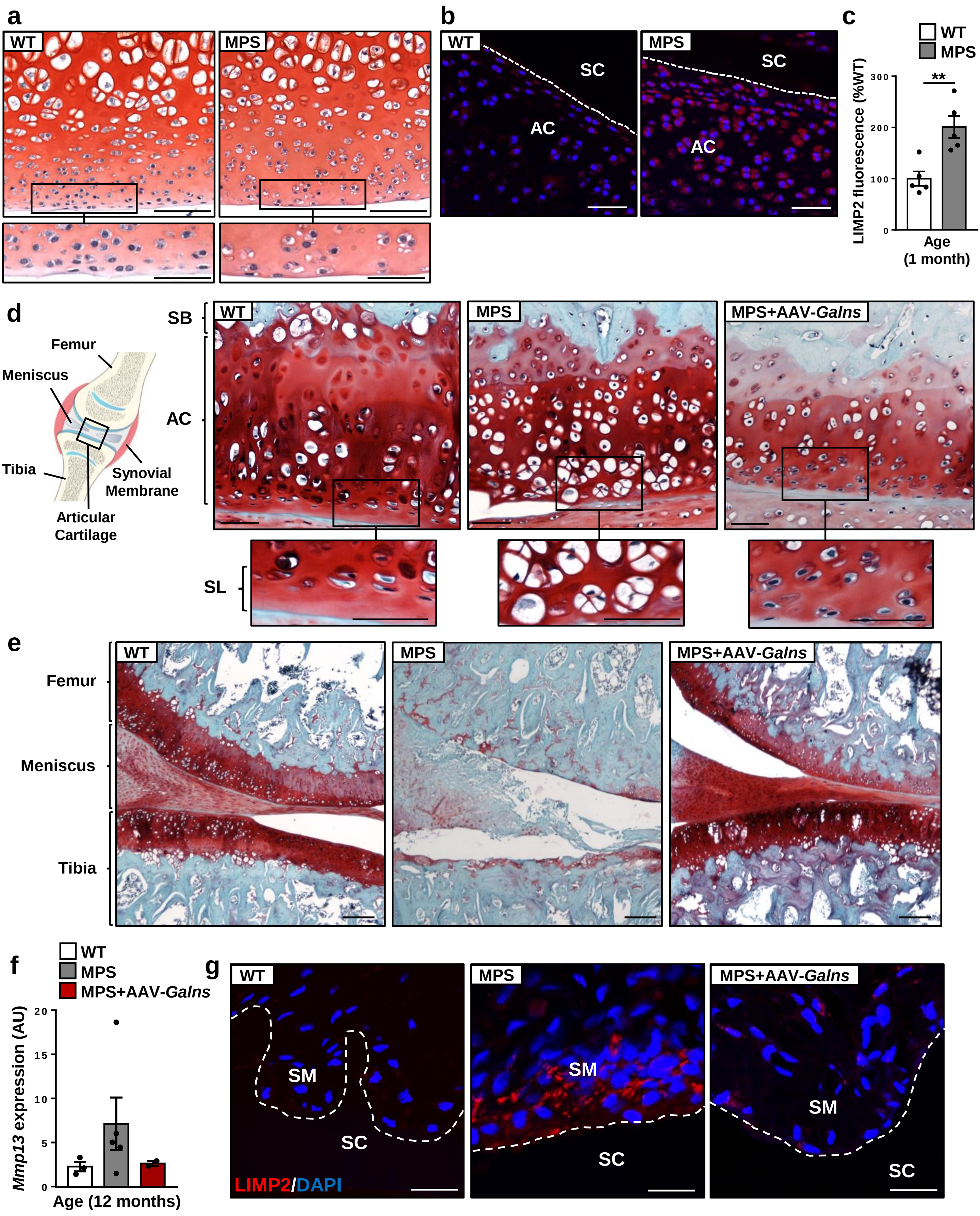

Supplementary Fig. 4. AAV9-*Galns* treatment ameliorates joint pathology. Four-week-old MPSIVA rats were treated with 6.67×10^{13} vg/kg AAV9-*Galns* vectors. **a** Safranin O staining of histological sections of tibial articular cartilage of 1-month-old WT and MPSIVA rats. Scale bars: 100µm; insets, 50µm. **b** LIMP2 immunohistochemical analysis of tibial articular cartilage sections of the same cohort as in (a). Lysosomal distension was detected in articular chondrocytes from MPSIVA rats. Scale bars: 50µm. AC, Articular Cartilage; SC, Synovial Cavity. **c** Quantification of LIMP2 mean fluorescence in articular cartilage of the same cohort as in (a) (n=5 animals/group). **d,e** At 12 months of age, histological analysis of articular cartilage (AC) sections from knee joints stained with Safranin O from WT, untreated and AAV9-*Galns*-treated MPSIVA rats showed AC chondrocyte hypertrophy only in untreated MPSIVA rats (d) (n=4 animals/group). Scale bars: 50µm; insets, 20µm. SB, subchondral bone; SL, superficial layer. Some untreated MPSIVA rats also showed complete loss of AC (e). Scale bars: 50µm. **f** Matrix metalloprotease 13 *(Mmp13)* gene expression was determined by qPCR in articular cartilage of 12-month-old WT (n=3), untreated (n=5) and AAV9-*Galns*-treated (n=2) MPSIVA rats. **g** LIMP2 immunohistochemistry of synovial membrane (SM) sections from 6-month-old WT, untreated and treated MPSIVA rats 5 months after AAV9-*Galns* delivery (n=3 animals/group). Scale bars: 50µm. Results are shown as mean±SEM. $^{**}P<0.01$ *vs.* WT rats.

Supplementary Fig. 5

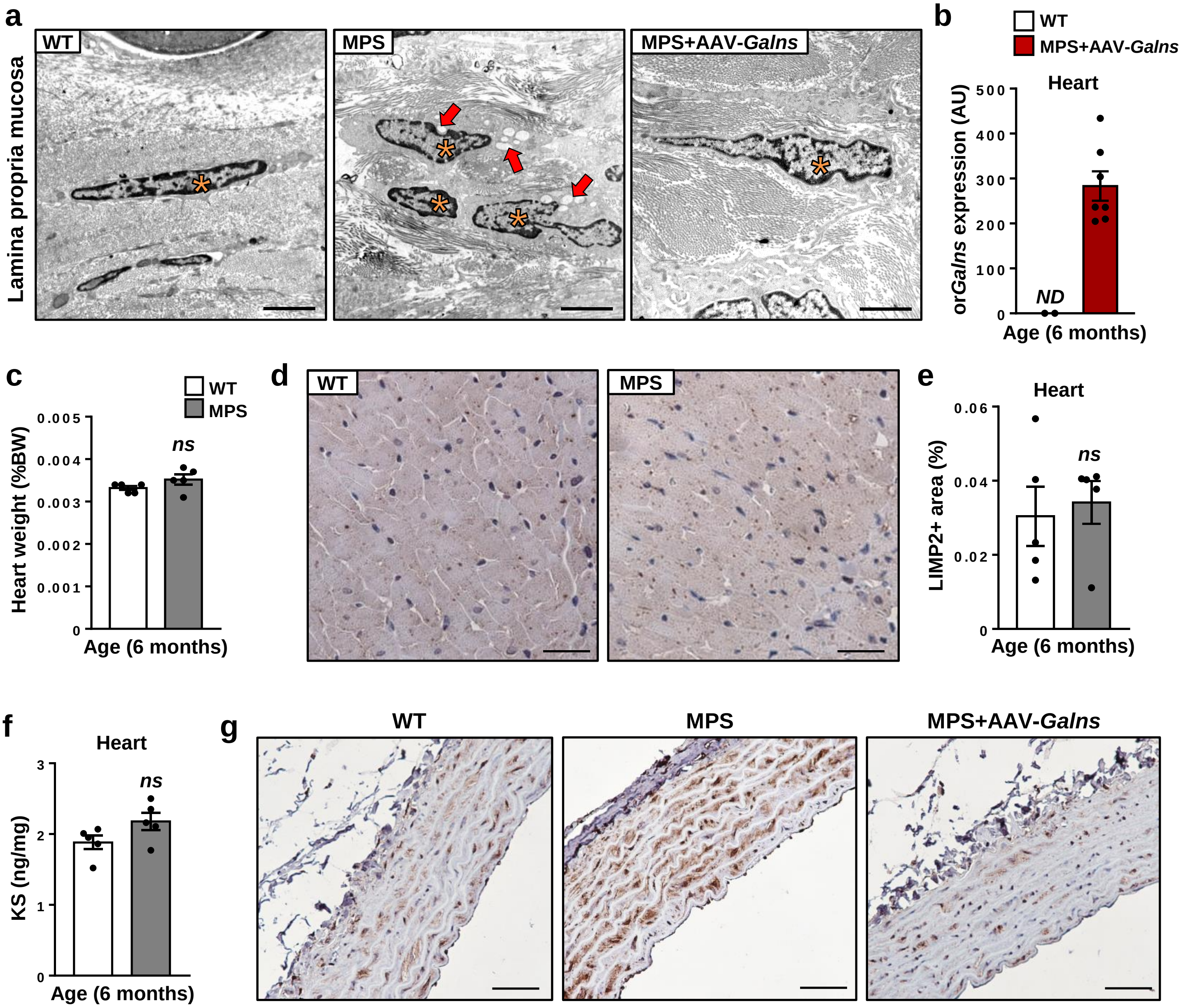

Supplementary Fig. 5. Effect of AAV9-*Galns* treatment in trachea and heart of MPSIVA rats. Four-week-old MPSIVA rats were treated with 6.67x10^{13} vg/kg AAV9-*Galns* vectors. **a** Five months after treatment, ultrastructural analysis of fibroblasts (asterisk) from lamina propria mucosa of the trachea from WT, untreated and AAV9-*Galns*-treated MPSIVA rats evidenced the presence of large electrolucent vacuoles (arrows) only in fibroblasts of untreated MPSIVA rats. Scale bars: 10µm. **b** Expression of optimized rat *Galns* (or*Galns)* in heart of 6-month-old WT (n=6) and AAV9-*Galns*-treated MPSIVA (n=7) rats. **c** Heart weight from 6-month-old WT and MPSIVA rats (n=5 animals/group). Results are presented as % of body weight (BW). **d** LIMP2 immunohistochemistry of myocardial sections from the same cohort as in (c). No lysosomal distension was detected in MPSIVA rats. **e**, Quantification of LIMP2+ area in heart sections analyzed in (d). **f** KS content in heart of the same cohort of animals as in (c). **g** LIMP2 immunohistochemistry of aorta sections from 6-month-old WT, untreated and AAV9-*Galns*-treated MPSIVA rats. Lysosomal distension can only be observed in smooth muscle fibers from aorta of MPSIVA rats. Results are shown as mean±SEM. *ND*, non-detected.
