# Supplementary material for: Treatment of skeletal and non-skeletal alterations of Mucopolysaccharidosis type IVA by AAV-mediated gene therapy
Source: Nat Commun. 2021 Sep 9;12:5343. doi: 10.1038/s41467-021-25697-y (PMC8429698; doi:10.1038/s41467-021-25697-y)
Supplement: Supplementary file 2 — Reporting Summary [file 41467_2021_25697_MOESM2_ESM.pdf]

Corresponding author(s): Fàtima Bosch

Last updated by author(s): Jul 15, 2021

Reporting Summary

Nature Research wishes to improve the reproducibility of the work that we publish. This form provides structure for consistency and transparency in reporting. For further information on Nature Research policies, see our Editorial Policies and the Editorial Policy Checklist.

Statistics

For all statistical analyses, confirm that the following items are present in the figure legend, table legend, main text, or Methods section.

n/a	Confirmed	
☐	☒	The exact sample size (*n*) for each experimental group/condition, given as a discrete number and unit of measurement
☐	☒	A statement on whether measurements were taken from distinct samples or whether the same sample was measured repeatedly
☐	☒	The statistical test(s) used AND whether they are one- or two-sided<br>*Only common tests should be described solely by name; describe more complex techniques in the Methods section.*
☒	☐	A description of all covariates tested
☒	☐	A description of any assumptions or corrections, such as tests of normality and adjustment for multiple comparisons
☐	☒	A full description of the statistical parameters including central tendency (e.g. means) or other basic estimates (e.g. regression coefficient) AND variation (e.g. standard deviation) or associated estimates of uncertainty (e.g. confidence intervals)
☐	☒	For null hypothesis testing, the test statistic (e.g. *F*, *t*, *r*) with confidence intervals, effect sizes, degrees of freedom and *P* value noted<br>*Give P values as exact values whenever suitable.*
☒	☐	For Bayesian analysis, information on the choice of priors and Markov chain Monte Carlo settings
☒	☐	For hierarchical and complex designs, identification of the appropriate level for tests and full reporting of outcomes
☒	☐	Estimates of effect sizes (e.g. Cohen's *d*, Pearson's *r*), indicating how they were calculated

Our web collection on statistics for biologists contains articles on many of the points above.

Software and code

Policy information about availability of computer code

Data collection	Fiji/ImageJ 1.53c, MicroView 3D Image Viewer & Analysis Tool 2.2, NIS Elements Advanced Research 2.20
Data analysis	GraphPad Prism 7, Microsoft Excel 2013

For manuscripts utilizing custom algorithms or software that are central to the research but not yet described in published literature, software must be made available to editors and reviewers. We strongly encourage code deposition in a community repository (e.g. GitHub). See the Nature Research guidelines for submitting code & software for further information.

Data

Policy information about availability of data

All manuscripts must include a data availability statement. This statement should provide the following information, where applicable:

- Accession codes, unique identifiers, or web links for publicly available datasets
- A list of figures that have associated raw data
- A description of any restrictions on data availability

The authors declare that all data supporting the findings of this study are available within the paper. Source data are provided with this paper.

Field-specific reporting

Life sciences study design

All studies must disclose on these points even when the disclosure is negative.

Sample size	No statistical methods were used to pre-determine sample sizes but we have extensive previous experience for determining experimental sample size and our sample sizes are similar to those reported in previous publications in the field (Ruzo et al., 2012; Haurigot et al., 2013; Ribera et al., 2015; Marcó et al., 2016; Motas et al., 2016; Roca et al., 2017)
Data exclusions	No data was excluded from the study
Replication	All the experiments represented in figures 1a, 1b, 1c, 1d, 1e, 2a, 2b, 2d, 2g, 3a, 3b, 3e, 4a, 4b, 4d, 5a, 5b, 5d, 5e, 6a, 6b, 6c, 6g and supplementary figures 1b, 1c, 1d, 1e, 1f, 1g, 1h, 1i, 2a, 2b, 2c, 2d, 2e, 2f, 2g, 2h, 2i, 3a, 3b, 3c, 3d, 3k, 4a, 4b, 4c, 4d, 4e, 4g, 5b, 5c, 5d, 5e, 5g were repeated twice. The rest of experiments could not be repeated due to the lack or reduced sample availability or elevated cost
Randomization	All animals were randomized to treatment versus control groups. WT, MPSIVA and AAV9-treated MPSIVA rats were housed all together after weaned from same parents in the same experimental conditions (diet, light and temperature)
Blinding	All experiments were performed under blinding conditions, except those experiments were rat phenotype was visuable indentificable to the investigator (grip strenght, body weight and naso-anal length measurements).

Reporting for specific materials, systems and methods

We require information from authors about some types of materials, experimental systems and methods used in many studies. Here, indicate whether each material, system or method listed is relevant to your study. If you are not sure if a list item applies to your research, read the appropriate section before selecting a response.

Materials & experimental systems

n/a	Involved in the study
☐	☒ Antibodies
☐	☒ Eukaryotic cell lines
☒	☐ Palaeontology and archaeology
☐	☒ Animals and other organisms
☒	☐ Human research participants
☒	☐ Clinical data
☒	☐ Dual use research of concern

Methods

n/a	Involved in the study
☒	☐ ChIP-seq
☒	☐ Flow cytometry
☒	☐ MRI-based neuroimaging

Antibodies

Antibodies used	For GFP and LIMP2 immunohistochemistry, goat anti-GFP (Abcam: ab6673, dil 1:300) or rabbit anti-LIMP2 (Novus Biologicals: NB400-129, dil 1:100) primary antibodies were used. Biotinylated Alexa Fluor® 488 anti-goat IgG (Life technologies: A11055, dil 1:100) or Alexa Fluor® 568 anti-rabbit IgG (Invitrogen: A10042, dil:1:100) were used as secondary antibodies.
Validation	Manufacturer provided validation of antibodies in rat samples

Eukaryotic cell lines

Policy information about cell lines

Cell line source(s)	HEK293 cells were kindly provided by Children's Hospital of Philadelphia (CHOP)
Authentication	The cell lines were not authenticated
Mycoplasma contamination	Our cell lines were tested for mycoplasma contamination and they were all confirmed to be negative
Commonly misidentified lines (See ICLAC register)	Not commontly misidentifies cell lines were used in the study

Animals and other organisms

Policy information about studies involving animals; ARRIVE guidelines recommended for reporting animal research

Laboratory animals	The knock-in rat model of the MPSIVA disease was generated using CRISPR/Cas9 technology. To this end, the human and Rattus norvegicus GALNS protein sequences were aligned and the position of the human Arg386Cys mutation corresponded to position 388 in the rat protein sequence (Arg388Cys). Thus, the C>T single nucleotide change at the position 1156 of the human Galns coding sequence (CDS) is equivalent to the C at the position 1162 of the rat Galns CDS. Briefly, two specific RNA guides, gRNA1: 5’-CCC ATA TTT TAT TAC CGT GGC A-3’ and gRNA2: 5’-TAC CGT GGC AAC ACA CTG ATG G-3’, were designed to target exon 11 and drive the Cas9

	double strand break close to the 1162C genomic position. A single strand donor DNA sequence was designed to introduce both the 1162C>T missense mutation and a new MboII restriction site, required for genotyping analysis of the offspring. Two homology arms were also included in the donor DNA to enable homologous recombination with the rat Galns genomic sequence. The gRNA, donor DNA, and Cas9 mRNA were microinjected into the pronucleus of one-cell Sprague–Dawley (SD) rat embryos. Male rats of 1-, 1.5-, 2-, 3-, 6- and 12-month-old were analyzed. For follow-up studies (body weigth and GALNS activity) male rats ranging from 21 days to 126 days of age were analyzed.
Wild animals	No wild animals were used in this study
Field-collected samples	No field collected samples were used in the study
Ethics oversight	All experimental procedures were approved by the Ethics Committee for Animal and Human Experimentation of the Universitat Autònoma Barcelona (UAB).

Note that full information on the approval of the study protocol must also be provided in the manuscript.
